# Vascular malperfusion and abruption are prevalent in placentas from pregnancies with congenital heart disease and not associated with cardiovascular risk

**DOI:** 10.1038/s41598-023-28011-6

**Published:** 2023-01-25

**Authors:** Marie Altendahl, Thalia Mok, Ekene Adimkpayah, Jeffrey Goldstein, Jeannette Lin, Yalda Afshar

**Affiliations:** 1 Division of Maternal Fetal Medicine, Department of Obstetrics and Gynecology, David Geffen School of Medicine at UCLA, Los Angeles, CA USA; 2 Division of Cardiology, Department of Medicine, Ahmanson/UCLA Adult Congenital Heart Disease Center, David Geffen School of Medicine at UCLA, Los Angeles, CA USA; 3 Department of Pathology and Laboratory Medicine, David Geffen School of Medicine at UCLA, Los Angeles, CA USA

**Keywords:** Reproductive biology, Cardiovascular biology

## Abstract

Congenital heart disease (CHD) in pregnancy is associated with an increased risk of adverse maternal, obstetric, and neonatal outcomes, plausibly through mechanisms involving abnormal placental development and function. This retrospective study aims to elucidate how maternal CHD influences placental health. Demographic and clinical information were collected via electronic medical record review, and placentas underwent histopathological evaluation. Fifty-three singleton pregnancies were included: 35 participants (66%) were classified as lower cardiovascular risk (modified World Health Organization Classification (mWHO) I, II, II-III), and 18 (34%) were classified as higher cardiovascular risk (mWHO III, IV). 12 participants (23%) had a fetus with small for gestational age (SGA). Maternal vascular malperfusion (53%) and placental abruption (11.6%) were common in this cohort, with prevalence above baseline risk. Participants at higher cardiovascular risk had higher rates of SGA (p = 0.04), subchorionic hematomas (p = 0.01) and birth weight:placental weight < 10th percentile (p = 0.04), but did not differ in rates of maternal vascular malperfusion (p = 0.15) compared to those at lower cardiovascular risk. In pregnancies with maternal CHD, SGA and histologic evidence of maternal vascular malperfusion and placental abruption were common, though patients at higher cardiovascular risk did not show evidence of worsened placental health compared to those at lower risk.

## Introduction

With advances in surveillance and medical and surgical intervention for congenital heart disease (CHD), there has been a substantial increase in reproductive-aged adults with CHD^1^. In pregnancy, alterations in normal cardiovascular changes of pregnancy put birthing persons with CHD at high risk for heart failure, arrhythmias, and thrombotic events due to increased hemodynamic stress^1–3^. Additionally, maternal CHD is associated with adverse pregnancy and neonatal outcomes, such as higher rates of preterm birth, small for gestational age (SGA) birth weights, and neonatal mortality^2,3^. Although it remains unclear what mediates the relationship between maternal CHD and these adverse pregnancy and neonatal outcomes, it is biologically plausible that altered placental development and function, as a conduit of the feto-maternal system, drive these outcomes^4^.

The development of the placenta, a structure imperative for transporting oxygen and nutrients to fetus, is heavily influenced by both maternal and fetal health^5^. Maternal cardiovascular risk factors such as obesity, diabetes, and hypertension have been identified to negatively affect placental development and function. Maternal obesity increases inflammation of placental tissues, alters expression and activity of the lipid transporters in the placenta, and creates excess production of reactive oxygen species^6,7^. These changes in placental function seen in maternal obesity, can result in poor fetal growth and even fetal demise^8^. However, while it is clear that maternal cardiovascular health influences placental health, there are very few studies investigating the effects of maternal CHD on placental function. Maternal CHD is thought to restrict cardiac output and decreas blood flow to the placenta, resulting in maternal vascular malperfusion^4,8^. Evidence of maternal vascular malperfusion on histopathological evaluation has been shown in pregnancies with maternal CHD^4^.

Maternal cardiovascular health appears to influence placental vascular development and the adverse birthing outcomes seen in pregnancies complicated by maternal CHD may be placentally driven. In this study, we aim to investigate the relationship between maternal CHD cardiovascular risk and placental health. We hypothesize that pregnancies with higher cardiovascular risk will have greater evidence of maternal vascular malperfusion on histopathologic placenta examination.

## Results

For initial analysis, 134 pregnancies with maternal CHD that received care with our facility’s cardio-obstetrics team were identified. A total of 81 pregnancies were excluded due to not having placental pathology data available. The remaining 53 participants were included in analyses. The average maternal age of participants was 29.8 years (range: 18–41). 27 (50.9%) participants had vaginal births, 16 (30.2%) had cesarean births, 6 (11.3%) had forceps-assisted vaginal births, and 4 (7.6%) had vacuum-assist births. 12 participants (22.6%) had fetuses with SGA. Additional demographic and clinical characteristics can be found in Table 1.

**Table 1:** Summary of maternal demographics, maternal clinical characteristics, pregnancy outcomes, and neonatal outcomes by classification of cardiovascular risk. *p<0.05.

Demographics	Total (N = 53)	mWHO I, II, II-III (n = 35)	mWHO III, IV (n = 18)	p-value
	Median (IQR)	Median (IQR)	Median (IQR)	
Age	30 (25–35)	31(25–35)	28.5 (24–32)	0.93

Demographics	n (%)	n (%)	n (%)	p-value
Race/ethnicity				0.60
American Indian or Alaskan Native	1 (1.9)	1 (2.9)	0 (0.0)	
Asian	5 (9.4)	5 (14.3)	0 (0.0)	
Black	2 (3.8)	1 (2.6)	1 (5.6)	
Hispanic	13 (24.5)	8 (22.9)	5 (27.8)	
White	27 (50.9)	17 (48.6)	10 (55.6)	
Other	5 (9.4)	3 (8.6)	2 (11.1)	

Co-morbidities	n (%)	n (%)	n (%)	p-value
Hx of smoking	2 (3.8)	1 (2.9)	1 (5.6)	1.00
Diabetes	2 (3.8)	1 (2.9)	1 (5.6)	1.00
Chronic hypertension	6 (11.3)	1 (2.9)	5 (27.8)	0.01*
Asthma	3 (5.7)	2 (5.7)	1 (5.6)	1.00
Thyroid condition	4 (7.6)	1 (2.9)	3 (16.7)	0.11
Autoimmune disorders	1 (1.9)	1 (2.9)	0 (0.0)	1.00

Pregnancy outcomes	Median (IQR)	Median (IQR)	Median (IQR)	p-value
Gestational age at delivery (weeks)	38.1 (36.1–39.1)	39 (36.6–39.1)	37.1 (35.9–38.3)	0.02*
Estimated blood loss (ml)	350 (200–700)	350 (200–686)	430 (250–800)	0.57

Pregnancy outcomes	n (%)	n (%)	n (%)	p-value
Hypertensive disorders of pregnancy	15 (28.3)	9 (25.7)	6 (33.3)	0.75
Gestational diabetes mellitus	5 (9.4)	4 (11.4)	1 (5.6)	0.65
Fetal growth restriction	10 (18.9)	6 (17.1)	4 (22.2)	0.72
Postpartum hemorrhage	6 (11.3)	5 (14.3)	1 (5.6)	0.65
Peripartum infection	9 (17.0)	4 (11.4)	5 (27.8)	0.25
Maternal ICU admission	4 (7.6)	0 (0.0)	4 (22.2)	0.01*

Mode of birth	n (%)	n (%)	n (%)	p-value
Vaginal birth	27 (50.9)	19 (54.3)	8 (44.4)	0.40
Cesarean birth	16 (30.2)	11 (31.4)	5 (27.8)	
Forceps-assisted birth	6 (11.3)	4 (11.4)	2 (11.1)	
Vacuum-assisted birth	4 (7.6)	1 (2.9)	3 (16.7)	

Neonatal outcomes	Median (IQR)	Median (IQR)	Median (IQR)	p-value
Birth weight (g)	2805 (2320–3195)	3060 (2390–3210)	2560 (1972–2690)	0.002*
1-min APGAR	8 (7–9)	8 (7–9)	8 (6–8)	0.06
5-min APGAR	9 (9–9)	9 (9–9)	9 (8–9)	0.17

Neonatal outcomes	n (%)	n (%)	n (%)	p-value
Small for gestational age	12 (22.6)	5 (14.3)	7 (38.9)	0.04*
Large for gestational age	0 (0.0)	0 (0.0)	0 (0.0)	N/A
NICU admission	17 (32.1)	9 (25.7)	8 (44.4)	0.17
Respiratory distress syndrome	8 (15.1)	3 (8.6)	5 (27.8)	0.10
Transient tachypnea of newborn	0 (0.0)	0 (0.0)	0 (0.0)	N/A
Necrotizing entercolitis	0 (0.0)	0 (0.0)	0 (0.0)	N/A
IVH	1 (1.9)	1 (2.9)	0 (0.0)	1.00
Hypoxic ischemic encephalopathy	0 (0.0)	0 (0.0)	0 (0.0)	N/A
Sepsis	0 (0.0)	0 (0.0)	0 (0.0)	N/A
Neonatal death	1 (1.9)	0 (0.0)	1 (5.6)	0.34

**Figure 1:**
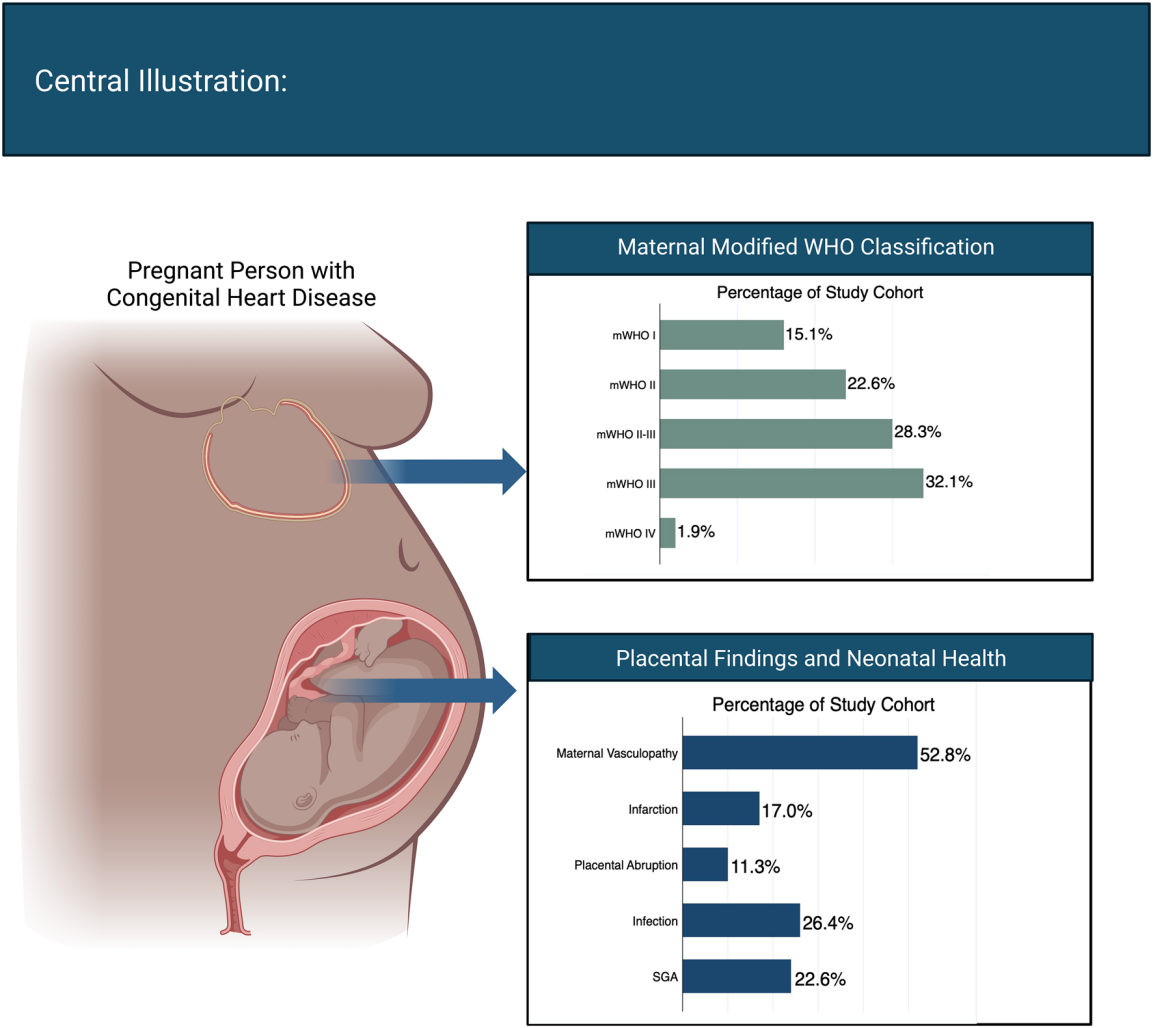
Central illustration.

There was a wide range of congenital cardiac defects seen in our population (Fig. 1). The most common diagnoses included aortic valvular disease (17.0%), Tetralogy of Fallot or pulmonary valve disease (17.0%), intracardiac shunt lesion (13.2%), and transposition of the great arteries (11.3%). Participants were classified using the modified World Health Organization (mWHO) classification: mWHO I: 8 (15.1%) participants, mWHO II: 12 (22.6%) participants, mWHO II-III: 15 (28.3%) participants, mWHO III: 17 (32.1%) participants, mWHO IV: 1 (1.9%) participant. 35 participants (66.0%) were classified as being at lower cardiovascular risk (mWHO I, II, II-III) and 18 (34.0%) were classified as higher cardiovascular risk (mWHO III, IV). Further details regarding participant congenital cardiac diagnosis, and associated mWHO classification are listed in Table 2.

**Table 2:** Description of congenital heart disease (CHD) by mWHO classification in our total study population.

mWHO classification	Congenital heart disease diagnosis	n (%)
mWHO I (N = 8)	Uncomplicated pulmonary stenosis	0 (0.0)
	Uncomplicated patent ductus arteriosis	1 (1.9)
	Uncomplicated mitral valve prolapse	1 (1.9)
	Repaired simple lesions (ASD, VSD, PDA)	5 (9.4)
	isolated atrial or ventricular ectopic beats	0 (0.0)
	Other “low risk” condition	1 (1.9)
mWHO II (N = 12)	Unoperated ASD or VSD	2 (3.8)
	Repaired tetralogy of fallot	6 (11.3)
	Cardiac arrhythmia	0 (0.0)
	Other “low risk” condition	4 (7.5)
mWHO II-III (N = 15)	Mild left ventricular impairment	0 (0.0)
	Hypertrophic cardiomyopathy	0 (0.0)
	Valvular heart disease not considered mWHO I or IV	8 (15.1)
	Marfan syndrome without aortic dilation	1 (1.9)
	Aortic disease without dilation	0 (0.0)
	Repaired coarctation	4 (7.5)
	Other “moderate risk” condition	2 (3.8)
mWHO III (N = 17)	Mechanical valve	2 (3.8)
	Systemic right ventricle	0 (0.0)
	Fontan circulation	4 (7.5)
	Cyanotic heart disease	6 (11.3)
	Other complex CHD	4 (7.5)
	Marfan syndrome with aortic dilation 45–50 mm	1 (1.9)
	Other “high risk” condition	0 (0.0)
mWHO IV (N = 1)	Pulmonary arterial hypertension	1 (1.9)
	Severe systemic ventricular dysfunction	0 (0.0)
	Previous peripartum cardiomyopathy	0 (0.0)
	Severe mitral stenosis or aortic stenosis	0 (0.0)
	Aortic dilation > 50 mm	0 (0.0)
	Native severe coarctation	0 (0.0)
	Other “high risk” condition	0 (0.0)

Participants at higher cardiovascular risk (mWHO III, IV) were more likely to have chronic hypertension (p = 0.01), be delivered at earlier gestational ages (p = 0.02), have lower birth weights (p = 0.002), higher prevalence of SGA (p = 0.04), and greater rates of maternal intensive care unit (ICU) admission (p = 0.01) compared to those at lower cardiovascular risk (mWHO I, II, II-III). There were no differences in mode of delivery (vaginal, cesarean, forceps-assisted, or vacuum assisted) between the mWHO I, II, II-III and mWHO III, IV cohorts (p = 0.40). All other demographic, clinical comorbidities, and pregnancy and neonatal outcomes were similar between the mWHO I, II, II-III and mWHO III, IV cohorts (Table 1, Fig. 1).

53 placentas were reviewed by pathology after birth and the frequencies of placental histopathologic findings are shown in Table 3. 28 placentas (52.8%) showed histopathologic evidence of maternal vascular malperfusion. The most common findings of maternal vascular malperfusion were infarction (17%), subchorionic hematoma (15.1%), and placental abruption (11.6%). 11 (20.8%) placentas had chorioamnionitis and 4 (7.6%) had deciduitis.

**Table 3:** Summary of abnormal placental findings seen on histopathological examination.

Placental characteristics	Mean, SD (range)
Placental weight (g)	411.7, 110 (201–679)

	n (%)
BW:PW < 3	2 (3.8)
BW:PW < 10	3 (5.7)
Thrombosis	5 (9.4)
Infarction	9 (17.0)
Chorangiosis	3 (5.7)
Hypomature villus	5 (9.4)
Maternal vascular malperfusion	28 (52.8)
Subchorionic hematoma	8 (15.1)
Placenta abruption	6 (11.3)
Chorioamnionitis and deciduitis	14 (26.4)
Cord abnormalities	7 (13.2)
Cord insertion abnormalities	6 (11.3)

Participants showed few differences in placental histopathologic findings based on modified WHO classification risk (Table 4). Both mWHO I, II, II-III and mWHO III, IV cohorts had similar rates of birth weight:placental weight < 3rd percentile (p = 0.11), evidence of thrombosis (p = 0.32), infarction (p = 0.47), chorangiosis (p = 1.00), hypomature villus (p = 1.00), placental abruption (p = 0.40), chorioamnionitis (p = 0.37) and deciduitis (p = 1.00), and cord abnormalities (p’s > 0.34). Further, there was no difference in evidence of maternal vascular malperfusion (p = 0.15) between these cohorts. Notably, participants at higher cardiovascular risk did have higher rates of birth weight:placental weight < 10th percentile (p = 0.04) and subchorionic hematomas (p = 0.01) compared to participants at lower cardiovascular risk.

**Table 4:** Placental histopathological findings in pregnancies by cardiovascular risk using modified WHO classification. *p<0.05.

	mWHO I, II, II-III (n = 35) n (%)	mWHO III, IV (n = 18) n (%)	P-value
BW:PW < 3	0 (0.0)	2 (11.1)	0.11
BW:PW < 10	0 (0.0)	3 (16.7)	0.04*
Thrombosis	2 (5.7)	3 (16.7)	0.32
Infarction	5 (14.3)	4 (22.2)	0.47
Chorangiosis	2 (5.7)	1 (5.6)	1.00
Hypomature villus	3 (8.6)	2 (11.1)	1.00
Maternal vascular malperfusion	16 (45.7)	12 (66.7)	0.15
Subchorionic hematoma	2 (5.7)	6 (33.3)	0.01*
Placental abruption	3 (8.6)	3 (16.7)	0.40
Chorioamnionitis and deciduitis	8 (22.9)	6 (33.3)	0.52
Hypocoiled cord	2 (5.7)	0 (0.0)	0.54
Hypercoiled cord	3 (8.6)	1 (5.6)	1.00
Single umbilical artery	0 (0.0)	1 (5.6)	0.34
Cord insertion abnormalities	4 (11.4)	2 (11.1)	1.00

Similar findings were seen between cohorts when defining “low cardiovascular risk” as mWHO I, II and “high cardiovascular risk” as mWHO II-III, III, IV (Supplemental Table 3). No differences were seen in placental histopathologic findings between mWHO I, II and mWHO II-III, III, IV cohorts.

## Discussion

Placentas of 53 birthing persons with congenital heart disease underwent histopathological evaluation. The most common findings were maternal vascular malperfusion (53%) and histologic evidence of infection (26%). Interestingly, there were high rates of infarction (17%) and placental abruption (11%) above baseline risk. SGA (23%) was also a common finding in this cohort of pregnancies complicated by maternal CHD. When comparing placental findings by mWHO classification of cardiovascular risk, there were few differences in histopathological findings between those at higher cardiovascular risk compared to lower cardiovascular risk, though higher cardiovascular risk was associated with higher prevalence of SGA. Importantly, higher cardiovascular risk (mWHO III, IV) was not associated with evidence of maternal vascular malperfusion.

Proper placental development is heavily influenced by maternal cardiovascular factors^9^. During pregnancy, alterations in the maternal vascular system initiates remodeling of maternal spiral arteries to increase oxygen and nutrient flow to the developing fetus^10^. Biological plausibility about placental development in maternal cardiovascular development could result from aberrant angiogenesis signaling, response, and subsequent differential vascular perfusion with neonatal sequalae. Howell et al. showed that maternal obesity results in downregulation of placental VEGF/Flt signaling resulting in decreased placental angiogenesis, placental hypoperfusion, and increased risk for pregnancy complications like SGA and pre-eclampsia^9^. Additionally, Wu et al. found that placentas from pregnancies complicated by maternal CHD, arrhythmia, cardiomyopathy, connective tissue disease, acquired valvular disease, ischemic heart disease, or vascular disease commonly showed evidence of vascular abnormalities^4^. Within the maternal CHD cohort, Wu et al. found no differences in anatomic, infectious, inflammatory, or vascular pathology based on anatomic or physiologic classification of CHD^4^. Our study similarly found high rates of SGA in pregnancies affected by maternal CHD, particularly in participants at higher cardiovascular risk. Additionally, maternal vascular malperfusion was common in our cohort, though few differences were seen in rates of histopathologic evidence of maternal vascular malperfusion based on cardiovascular risk. This finding was surprising, as we hypothesized that individuals with higher risk CHD (mWHO III, IV) have decreased blood flow and oxygenation to the placenta, resulting in greater evidence of maternal vascular malperfusion on histology and subsequent poorer fetal growth.

Our results may be limited by the wide variety of congenital cardiac defects seen in our cohort including: intracardiac shunts, aortic valve disease, mitral stenosis, mitral regurgitation and/or prolapse, Tetralogy of Fallot, pulmonary valve disease, transposition of the great arteries, single ventricle physiology, double outlet right ventricle, pulmonary hypertension, Marfan syndrome, Ebstein anomaly, and other cardiac defects. Although we estimated the cardiovascular risk of maternal CHD using the mWHO classification, the heterogeneity in our cohort possibly confounded our results as various cardiac defects may impact placental development in different ways. Instead, a more appropriate way to assess maternal CHD severity is not through anatomical classification, but rather looking at more functional variables such as maternal hypoxia which more directly relate to cardiac function in pregnancy. Additionally, our results may be confounded by selection bias given that other high-risk co-morbidities may have led to placental pathology collection in our patients. Our study institution currently utilizes a standardized protocol to collect placentas from all pregnancies impacted by maternal CHD, however placentas obtained prior to 2017 were collected based on the discretion of the delivering physician. In patients delivered prior to 2017, other health factors besides CHD may have led to placental collection. As such, our patient population may be skewed towards a “higher-risk” population in which other co-morbidities may have impacted placental development and function. Further, although evidence of maternal vascular malperfusion was common in our cohort, we may not have seen differences in maternal vascular malperfusion by CHD cardiovascular risk as our study examined placental pathology at birth and not placental function during pregnancy. It is difficult to assess when placental pathology arose during pregnancy and how that pathology influenced placental function though out pregnancy. In future prospective studies should assess placental function and development during gestation and pregnancy by interrogating growth, vascular parameters, and Doppler indices in this population with CHD.

The prevalence of placental abruption in our cohort is strikingly above a baseline rate of 0.6–1%^11^. Placental abruption, or a premature separation of a previously normally implanted placenta, occurs through unknown mechanisms outside of the coup-countercoup phenomena in trauma and with hypertensive diseases of pregnancy. However, it is well documented that an obstetrical history of placental abruption history has been correlated to future coronary heart disease risk, including both hemorrhagic and ischemic stroke^12,13^. These findings raise the consideration for a common abnormal vascular etiology related to both the biology underlying the abruption and the subsequent vascular event.

In summary, our study indicates that in pregnancies with maternal CHD, placental abruption, and histologic evidence of maternal vascular malperfusion are common. Notably, greater cardiovascular risk did not show evidence of poorer placental health compared to those with lower cardiovascular risk.

## Methods

In this retrospective study, pregnant people with known congenital heart disease delivered at the University of California, Los Angeles Ronald Reagan Hospital between April 23rd, 2016 and November 1st, 2021 were eligible, including participants with surgical correction of congenital heart disease. Participants who had acquired heart disease, terminated their pregnancy, did not deliver at our institution, or those without placental specimen collection were excluded. The study was approved by the Institutional Review Board (IRB), University of California, Los Angeles ((UCLA) IRB#17-000778) and informed consent was waived given the retrospective nature of the study. All study experiments were performed in accordance with the Institutional Review Board guidelines.

### Demographic and clinical data

Baseline demographic and clinical data were collected via electronic medical record review. Maternal health data included age, BMI, race/ethnicity, tobacco use, diabetes, chronic hypertension, asthma, thyroid, and autoimmune disorders. Classification of cardiovascular risk was estimated using the modified WHO (mWHO) classification system^3^. Lower risk CHD included mWHO I, mWHO II, and mWHO II-III. Higher risk CHD included mWHO III and mWHO IV. Birthing outcomes included gestational age at delivery, mode of delivery, neonatal birthweight, and SGA. SGA infants were defined by birthweight percentiles using the WHO growth standards^14^.

### Placental collection and processing

Per standardized clinical practice at the study institution initiated in 2017, placentas from pregnancies affected by maternal congenital heart disease were collected and sent to pathology for gross and microscopic histopathologic examination at birth. Prior to 2017, indication for placental pathology was up to the discretion of the delivering physician. The placental size was measured, and trimmed weight was recorded. All placentas were fixed in 10% buffered formalin. Sections submitted included two sections of umbilical cord, two sections of membrane, three full thickness sections of grossly normal appearing placenta from the chorionic plate to the basal plate and additional submitted sections of any grossly abnormal placenta. Sections underwent routine processing, were paraffin-embedded, sectioned at 3 to 5 µm and stained with hematoxylin and eosin. The pathologists categorized the histopathologic lesions according to the Amsterdam criteria^15^. Placenta histopathologic findings of interest included placental weight and evidence of thrombosis, infarction, chorangiosis, hypomature villi, subchorionic hematoma, placental abruption, chorioamnionitis, and deciduitis. Placentas were classified as having “maternal vascular malperfusion” if there was evidence of any of the following: thrombosis, infarction, chorangiosis, hypomature villi, subchorionic hematoma, and/or placental abruption. Cord abnormalities included hypocoiled cords, hypercoiled cords, or presence of a single umbilical artery. Cord insertion abnormalities included marginal or velamentous insertion. Of note, some pathological terms were collapsed within thrombosis, hypomature villus, and subchorionic hematoma (see Supplemental Table 1).

### Statistical analysis

A total of 53 participants were included in analyses. Chi square analysis, Fisher’s exact test, and Student’s T-test were used to determine statistical differences in demographic and clinical characteristics between low-risk CHD participants and high-risk CHD. Chi-square analysis and Fisher’s exact test were used to detect differences in placental pathology findings between participants low-risk CHD and high-risk CHD. Data were presented with means with standard deviations, medians with interquartile ranges, and counts with percentages, as appropriate. Statistical analyses were performed using STATA, and statistical significance was set at a p-value of < 0.05.

## Supplementary Information

Supplementary Table S1.

Supplementary Table S2.

Supplementary Table S3.

## Data Availability

The datasets generated during and/or analyzed during the current study are available from the corresponding author on reasonable request.
