# Supplementary material for: Vascular malperfusion and abruption are prevalent in placentas from pregnancies with congenital heart disease and not associated with cardiovascular risk
Source: Sci Rep. 2023 Jan 25;13:1439. doi: 10.1038/s41598-023-28011-6 (PMC9876959; doi:10.1038/s41598-023-28011-6)
Supplement: Supplementary file 1 — Supplementary Table S1. [file 41598_2023_28011_MOESM1_ESM.pdf]

Supplemental Table 1. Pathological terms that were collapsed into subheadings.

**Pathology term**	**Included pathology**
Thrombosis	Intervillous fibrin clot, mature chorionic villi with focal thrombus, thrombohematoma, retroamniotic blood clot, basal decimal fibrinoid necrosis with thrombosis
Hypomature villus	Distal villous hypoplasia, chorionic villi with focal dysmaturity, delayed villous maturation
Subchorionic hematoma	Subchorionic hemorrhage, extensive subchorionic organizing hematomas, intervillous hematoma, placental hematoma, subchorionic thrombohematoma, subchorionic hematoma
