# Supplementary material for: Vascular malperfusion and abruption are prevalent in placentas from pregnancies with congenital heart disease and not associated with cardiovascular risk
Source: Sci Rep. 2023 Jan 25;13:1439. doi: 10.1038/s41598-023-28011-6 (PMC9876959; doi:10.1038/s41598-023-28011-6)
Supplement: Supplementary file 2 — Supplementary Table S2. [file 41598_2023_28011_MOESM2_ESM.docx]

|  | Total (N=53) | mWHO I, II (n=20) | mWHO II-III, III, IV (n=33) |  |
| --- | --- | --- | --- | --- |
| Demographics | Median (IQR) | Median (IQR) | Median (IQR) | p-value |
| Age | 30 (25-35) | 30.5 (25.5-35) | 30 (24-34) | 0.80 |
| Demographics | n (%) | n (%) | n (%) | p-value |
| *Race/Ethnicity* |  |  |  | 0.84 |
| American Indian or Alaskan Native | 1 (1.9) | 0 (0.0) | 1 (3.1) |  |
| Asian | 5 (9.4) | 3 (15.0) | 2 (6.1) |  |
| Black | 2 (3.8) | 1 (5.0) | 1 (3.0) |  |
| Hispanic | 13 (24.5) | 5 (25.0) | 8 (24.2) |  |
| White | 27 (50.9) | 10 (50.0) | 17 (51.5) |  |
| Other | 5 (9.4) | 1 (5.0) | 4 (12.1) |  |
| Co-morbidities | n (%) | n (%) | n (%) | p-value |
| Hx of Smoking | 2 (3.8) | 1 (5.0) | 1 (3.1) | 1.00 |
| Diabetes | 2 (3.8) | 0 (0.0) | 2 (6.1) | 0.52 |
| Chronic Hypertension | 6 (11.3) | 1 (5.0) | 5 (15.2) | 0.39 |
| Asthma | 3 (5.7) | 2 (10.0) | 1 (3.0) | 0.55 |
| Thyroid Condition | 4 (7.6) | 1 (5.0) | 3 (9.1) | 1.00 |
| Autoimmune Disorders | 1 (1.9) | 1 (5.0) | 0 (0.0) | 0.38 |
| Pregnancy Outcomes | Median (IQR) | Median (IQR) | Median (IQR) | p-value |
| Gestational Age at Delivery (weeks) | 38.1 (36.1-39.1) | 38.9 (37.9-39.1) | 37.1 (35.9-39.0) | 0.07 |
| Estimated Blood Loss (ml) | 350 (200-700) | 350 (250-608) | 410 (200-800) | 0.25 |
| Pregnancy Outcomes | n (%) | n (%) | n (%) | p-value |
| Hypertensive disorders of pregnancy | 15 (28.3) | 6 (30.0) | 9 (27.3) | 0.83 |
| Gestational Diabetes Mellitus | 5 (9.4) | 2 (10.0) | 3 (9.1) | 1.00 |
| Fetal Growth Restriction | 10 (18.9) | 4 (20.0) | 6 (18.2) | 1.00 |
| Postpartum Hemorrhage | 6 (11.3) | 1 (5.0) | 5 (15.5) | 0.39 |
| Peripartum Infection | 9 (17.0) | 2 (10.0) | 7 (21.2) | 0.46 |
| Maternal ICU Admission | 4 (7.6) | 0 (0.0) | 4 (12.1) | 0.29 |
| Mode of Birth | n (%) | n (%) | n (%) | p-value |
| Vaginal Birth | 27 (50.9) | 12 (60.0) | 15 (45.5) | 0.44 |
| Cesarean Birth | 16 (30.2) | 6 (30.0) | 10 (30.3) |  |
| Forceps-assisted Birth | 6 (11.3) | 2 (10.0) | 4 (12.1) |  |
| Vacuum-assisted Birth | 4 (7.6) | 0 (0.0) | 4 (12.1) |  |
| Neonatal Outcomes | Median (IQR) | Median (IQR) | Median (IQR) | p-value |
| Birth Weight (g) | 2805 (2320-3195) | 3100 (2655-3260) | 2640 (2260-3051) | 0.02* |
| 1-minute APGAR | 8 (7-9) | 8 (7.5-8) | 8 (7-9) | 0.40 |
| 5-minute APGAR | 9 (9-9) | 9 (9-9) | 9 (8-9) | 0.21 |
| Neonatal Outcomes | n (%) | n (%) | n (%) | p-value |
| Small for Gestational Age | 12 (22.6) | 3 (15.0) | 9 (27.3) | 0.50 |
| Large for Gestational Age | 0 (0.0) | 0 (0.0) | 0 (0.0) | N/A |
| NICU Admission | 17 (32.1) | 4 (20.0) | 13 (39.4) | 0.23 |
| Respiratory Distress Syndrome | 8 (15.1) | 1 (5.0) | 7 (21.2) | 0.23 |
| Transient Tachypnea of Newborn | 0 (0.0) | 0 (0.0) | 0 (0.0) | N/A |
| Necrotizing Entercolitis | 0 (0.0) | 0 (0.0) | 0 (0.0) | N/A |
| IVH | 1 (1.9) | 1 (5.0) | 0 (0.0) | 0.38 |
| Hypoxic Ischemic Encephalopathy | 0 (0.0) | 0 (0.0) | 0 (0.0) | N/A |
| Sepsis | 0 (0.0) | 0 (0.0) | 0 (0.0) | N/A |
| Neonatal Death | 1 (1.9) | 0 (0.0) | 1 (3.1) | 1.00 |

**Supplemental Table 2:** Summary of maternal demographics, maternal clinical characteristics, pregnancy outcomes, and neonatal outcomes by classification of cardiovascular risk using the modified WHO classification
