# Supplementary material for: Vascular malperfusion and abruption are prevalent in placentas from pregnancies with congenital heart disease and not associated with cardiovascular risk
Source: Sci Rep. 2023 Jan 25;13:1439. doi: 10.1038/s41598-023-28011-6 (PMC9876959; doi:10.1038/s41598-023-28011-6)
Supplement: Supplementary file 3 — Supplementary Table S3. [file 41598_2023_28011_MOESM3_ESM.docx]

**Supplemental Table 3:** Placental histopathological findings in pregnancies by cardiovascular risk using modified WHO classification

|  | mWHO I, II (n=20)  n (%) | mWHO II-III, III, IV (n=33)  n (%) | P-value |
| --- | --- | --- | --- |
| BW:PW <3 | 0 (0.0) | 2 (6.1) | 0.52 |
| BW:PW <10 | 0 (0.0) | 3 (9.1) | 0.28 |
| Thrombosis | 2 (10.0) | 3 (9.1) | 1.00 |
| Infarction | 2 (10.0) | 7 (21.2) | 0.46 |
| Chorangiosis | 0 (0.0) | 3 (9.1) | 0.28 |
| Hypomature Villus | 2 (10.0) | 3 (9.1) | 1.00 |
| Maternal Vascular Malperfusion | 8 (40.0) | 20 (60.6) | 0.15 |
| Subchorionic Hematoma | 2 (10.0) | 6 (18.2) | 0.70 |
| Placental Abruption | 2 (10.0) | 4 (12.1) | 1.00 |
| Chorioamnionitis & Deciduitis | 5 (25.0) | 9 (27.3) | 1.00 |
| Hypocoiled cord | 0 (0.0) | 2 (6.1) | 0.52 |
| Hypercoiled cord | 1 (5.0) | 3 (9.1) | 1.00 |
| Single Umbilical Artery | 0 (0.0) | 1 (3.0) | 1.00 |
| Cord insertion Abnormalities | 2 (10.0) | 4 (12.1) | 1.00 |
